# Effect of Spatial Position in the Field of View on Dimensional Changes in Cone Beam Computed Tomography

**Published:** 2017-09

**Authors:** Mehrdad Panjnoush, Yasaman Kheirandish, Negar Zeini

**Affiliations:** 1 Associate Professor, Dental Research Center, Dentistry Research Institute, Tehran University of Medical Sciences, Tehran, Iran; Department of Oral and Maxillofacial Radiology, School of Dentistry, Tehran University of Medical Sciences, Tehran, Iran; 2 Assistant Professor, Department of Oral and Maxillofacial Radiology, School of Dentistry, Tehran University of Medical Sciences, Tehran, Iran; 3 Postgraduate Student, Department of Oral and Maxillofacial Radiology, School of Dentistry, Tehran University of Medical Sciences, Tehran, Iran

**Keywords:** Cone-Beam Computed Tomography, Dimensional Measurement Accuracy, Orientation, Spatial

## Abstract

**Objectives:**

This study aimed to assess the relation between dimensional changes and object location in the field of view (FOV) using cone beam computed tomography (CBCT).

**Materials and Methods:**

A custom-made phantom was fabricated from base plate wax. To analyze the accuracy of measurements in horizontal and longitudinal dimensions, aluminum squares (0.5 mm thickness, 10×10 mm dimensions) were constructed and placed in three levels (upper, middle, and lower) and five positions (central, right, left, anterior and posterior). This phantom was scanned using Asahi, Planmeca and NewTom CBCT systems. CBCT scans were measured three times by use of their corresponding software. Statistical analysis was performed using one-way ANOVA, post-hoc test and two-way ANOVA (P<0.05).

**Results:**

The differences between the mean horizontal dimensions of different systems were not significant (P=0.296). However, the differences between the mean longitudinal dimensions of different systems were significant (P=0.039). The differences between the different positions and the mean horizontal and longitudinal dimensions were significant (P<0.001, and P<0.001, respectively). The differences between the mean horizontal dimensions and different levels were not significant (P=0.51), but the differences between the mean longitudinal dimensions and different levels were significant (P<0.001). The interaction effect of level and position on the accuracy of horizontal and longitudinal measurements was significant (P<0.0001).

**Conclusions:**

We found statistically significant differences in most of our comparisons; however, these differences were not clinically significant. Therefore, CBCT could be an accurate device for measurement of dimensions of objects placed in different positions in the FOV.

## INTRODUCTION

Imaging plays an important role in clinical evaluation of dental structures. Two-dimensional Intraoral and extraoral radiographs have some limitations such as image distortion, superimposition of the underlying structures and misrepresentation of the object of interest. Numerous efforts have been performed with regard to three-dimensional (3D) imaging such as computed tomography (CT). Despite the advantages of CT, its application is limited in dentistry due to high cost, limited access and high radiation dose. The introduction of cone beam computed tomography (CBCT), especially in maxillofacial field, is a prelude to greater application of 3D imaging techniques. Increased use of CBCT in different fields of dentistry is unprecedented, and can lead to a revolution in maxillofacial imaging. Nowadays, the role of imaging techniques in providing guidance images for surgical procedures and application of third-party software programs has increased [1–7]. The advantages of CBCT include low cost, appropriate size of the device compared to the conventional CT machines, ease of use, providing 3D images of the areas of interest, rapid scanning procedure, low radiation dose and limitation of X-ray beam radiation to specific anatomical structures [8].

Various studies have evaluated the accuracy of CBCT measurements using different devices, and found conflicting results. Many studies used dry human skulls to evaluate the accuracy of CBCT measurements and reported excellent accuracy [9–15].

Although some reports found significant differences between the CBCT measurements and actual distances, these differences were not clinically significant [16–20]. Due to the fact that CBCT devices are designed for more accurate measurements and detection of details of anatomical structures, evaluation of the effective factors on the quality and accuracy of CBCT images is of great importance. Many factors, including field of view (FOV), beam quality and quantity, pixel size and rotation arc are effective on the accuracy and quality of CBCT images, and thereby can influence the image features such as noise, contrast, resolution and artifacts [2, 21]. FOV refers to the scan volume of a particular CBCT unit. It can be dependent on the detector size and shape, the beam projection geometry and the beam collimation ability. In fact, FOV could be defined as area of interest to be covered by the beam. On the other hand, collimation of the primary X-ray beam can limit the X-radiation exposure to the region of interest and determine the range of image quality based on each patient’s specific needs.

The shape of FOV (volume scan) can be either cylindrical or spherical [4]. To evaluate the quality and accuracy of CBCT scans, in most studies, the object was located at the center of FOV. However, the impact of placing the object in peripheral position has not yet been evaluated. Some studies have focused on the object position in the FOV and reported that some features can be influenced by this factor such as noise, artifacts and homogeneity [22–26]. However, to our knowledge, no study has evaluated the impact of the object spatial position in the FOV on the dimensional changes. When we mention dimensional changes, we mean the difference between CBCT images in comparison to actual dimensions.

On the other hand, different factors such as size of the jaw and the head position can lead to placement of patient’s jaw in different areas in the FOV. This study aimed to answer the following question: Do dimensional changes occur if the object is placed in higher or lower level than the center position in the FOV?

Given the fact that in every rotation of X-ray tube, different spatial points with different distances from the tube and detector are placed in the FOV, we assessed the relation between the dimensional changes and object spatial position in the FOV.

## MATERIALS AND METHODS

### Image phantom preparation

A custom-made phantom was fabricated from 2 cylinders of base plate wax (diameter: 8 cm; height: 4 cm) to mimic the space of the scanning volume.

**Fig. 1:**
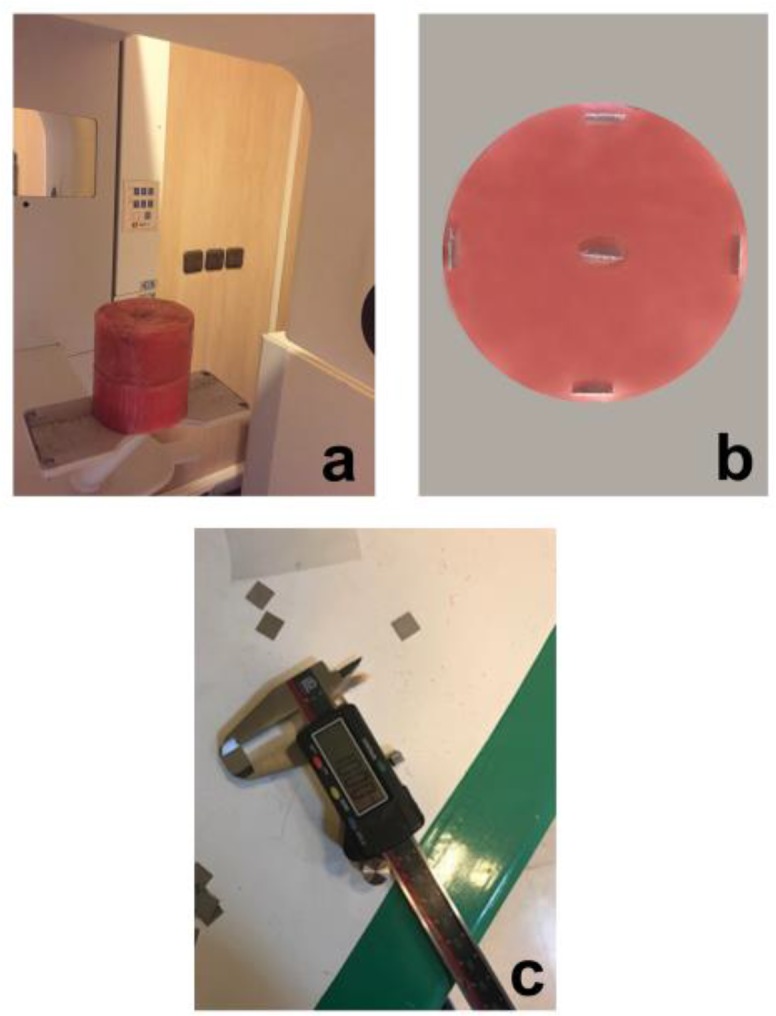
(a,b) the phantom used in this study; (c) digital caliper

To reconstruct the object, the aluminum squares (thickness: 0.5 mm, 10×10 mm dimensions) were fabricated using a wire cutting device (model AH840; Troop, Isfahan, Iran). This device had a precision of 0.01 mm, and was used for accurate cutting of metal. Following the cuttings, aluminum inserts were measured using a digital caliper. The exact measurements of aluminum inserts were 1cm×1cm (10.00mm). In order to evaluate the effect of object’s spatial position in the FOV, the aluminum inserts were placed into a cylindrical mold at three levels (upper, middle and lower) and five positions (center, right, left, anterior and posterior). Aluminum squares were placed inside a cylinder in a line. The aim of using two cylinders was the ease of aluminum insertion in the middle level (Fig. 1).

### CBCT systems

Phantom was scanned using three different CBCT systems:

1) Asahi (Alphard-3030 unit) (Asahi Roentgen Ind. Co., Ltd., Kyoto, Japan) operating at 4mA, 80kVp and 17s time in the FOV of 10×10 cm.
2) Planmeca (Promax 3D, Helsinki, Finland) operating at 12mA, 78kVp and 16s time in the FOV of 10×9 cm.
3) New Tom VG (QR s.r.l., Verona, Italy) operating at 6.65mA, 110kVp and 15s time in the FOV of 10×10 cm.

### Image analysis

For image reconstruction, the corresponding software was used. The sections were cut and coded by assigning a random number to each section.

**Fig. 2:**
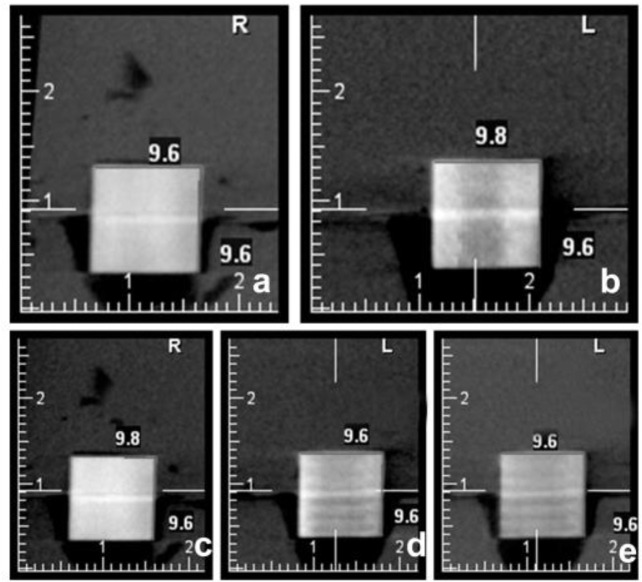
Cross-sectional images of CBCT scans: (a) left position; (b) center; (c) right position; (d) anterior position; (e) posterior position

Considering the fact that horizontal and longitudinal dimensions were measured in coronal and sagittal plans, measurement was done in each of the three dimensions in these sections. All the CBCT scans were evaluated by three observers (oral and maxillofacial radiologists who had sufficient experience) (Fig. 2).

### Data analysis

Statistical analysis was performed using STATA software version 12 (STATA Corporation, College Station, Texas, USA). One-way ANOVA was used to compare differences between systems, levels and positions. When the results of one-way ANOVA was significant, the post-hoc test was used for pairwise comparisons. Two-way ANOVA was used to analyze the Interaction between different levels and positions. P-values <0.05 were considered statistically significant.

## RESULTS

The inter-observer agreement for the horizontal dimension among the three observers was reported to be good, with Interclass correlation coefficient of 0.689. The difference was statistically significant (P<0.001). Additionally, Intraobserver agreement for horizontal dimension between different evaluation times was reported to be excellent, with intra-class correlation coefficient of 0.959. The difference was statistically significant (P<0.001).

### The accuracy of horizontal and longitudinal dimensions of markers according to the system:

**Fig. 3:**
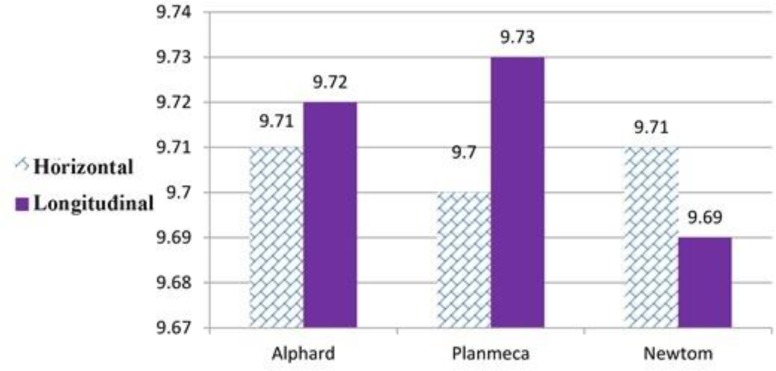
Effect of type of device on the mean horizontal and longitudinal dimensions (in millimeters)

The mean (standard deviation) values of horizontal and longitudinal dimensions were 9.71 (0.1) mm, and 9.72 (0.12) mm, respectively] for Asahi, 9.7 (0.12) mm, and 9.73 (0.12) mm, respectively for Planmeca, and 9.71 (0.15) mm, and 9.69 (0.14) mm, respectively for NewTom (Fig. 3). The differences between the horizontal dimensions measured by different systems were not significant (P=0.296, F= 0.296). The differences between the longitudinal dimensions measured by different devices were significant (P=0.039, F= 3.269) Table 1 shows the pairwise comparison between the mean difference of horizontal and longitudinal dimensions measured by different devices.

**Table 1.** Pairwise comparison of the P-values for the mean width and length in different systems

**Dimension**		**Asahi**	**Planmeca**	**NewTom**
Horizontal	Asahi	-	0.634	0.775
	Planmeca	-	-	0.447
	NewTom	-	-	-
Longitudinal	Asahi	-	0.3	0.13
	Planmeca	-	-	0.011
	NewTom	-	-	-

### The accuracy of horizontal and longitudinal dimensions of markers according to the position:

**Fig. 4:**
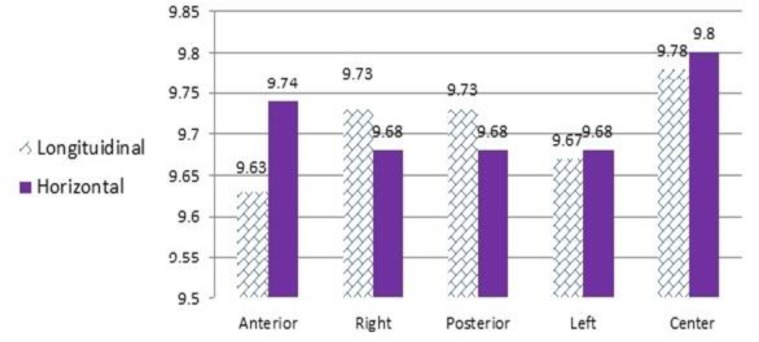
Effect of different positions on the mean horizontal and longitudinal dimensions (in millimeters)

The mean (standard deviation) values of horizontal and longitudinal dimensions were 9.63 (0.17) mm, and 9.74 (0.14) mm, respectively in the anterior position, 9.73 (0.11) mm and 9.68 (0.1) mm, respectively in the right position, 9.73 (0.14) mm and 9.68 (0.12) mm, respectively in the posterior position, 9.67 (0.12) mm and 9.68 (0.12) mm, respectively in the left position, and 9.78 (0.13) mm, and 9. 8 (0.14) mm, respectively in the central position (Fig. 4). The differences between the horizontal dimensions measured in different positions were significant (P<0.001, F= 19.842). In terms of horizontal dimension, there were significant differences between the different positions at each level. Central position had the least dimensional changes and the differences between the central and peripheral positions were significant (P<0.05). Regarding the peripheral position, the differences between the posterior and right positions were not significant (P>0.05). However, the differences between the other positions were significant (P<0.05). The differences between the longitudinal dimensions measured in different positions were significant (P<0.001, F= 14.591).

In terms of longitudinal dimensions, there were significant differences between the different positions at each level (P<0.05). Central position had the least dimensional changes and the difference between the central and peripheral positions was significant (P<0.05). In the peripheral position, the differences between the anterior and other positions were significant (P<0.05); the other differences were not statistically significant (P>0.05).

Table 2 shows pairwise comparisons of the mean differences of horizontal and longitudinal dimensions in different positions.

**Table 2.** Pairwise comparison of the P-values of the mean width and length according to different positions

**Dimension**		**Anterior position**	**Right position**	**Posterior position**	**Left position**	**Center**
**Horizontal**	Anterior position	-	<0.001	<0.001	0.016	<0.001
	Right position	-	-	-	0.003	0.005
	Posterior position	-	-	-	-	<0.001
	Left position	-	-	-	-	<0.001
	Center	-	-	-	-	-

**Longitudinal**	Anterior position	-	0.002	0.003	0.005	0.002
	Right position	-	-	0.898	0.797	< 0.001
	Posterior position	-	-	-	0.898	<0.001
	Left position	-	-	-	-	< 0.001
	Center	-	-	-	-	-

### The accuracy of horizontal and longitudinal dimensions of markers according to the level:

**Fig. 5:**
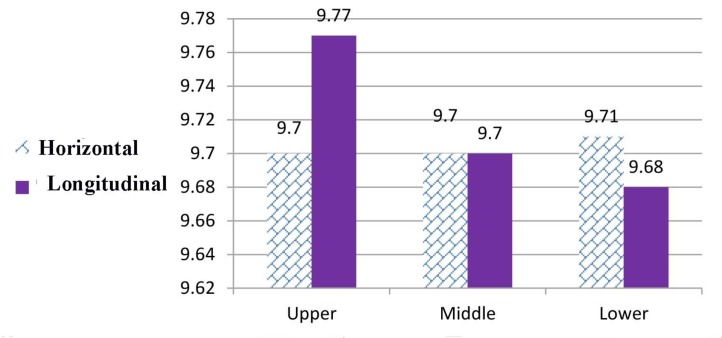
Effect of different levels on the mean horizontal and longitudinal dimensions (in millimeters)

The mean (standard deviation) values of horizontal and longitudinal dimensions were 9.7 (0.12) mm, and 9. 77 (0.13) mm, respectively in the upper level, 9.7 (0.13) mm and 9. 7 (0.12) mm, respectively in the middle level, and 9.71 (0.13) mm and 9. 68 (0.12) mm, respectively in the lower level (fig. 5).

The differences between the mean horizontal dimensions in different levels were not significant (P=0.51, F= 0.66). The differences between the mean longitudinal dimensions measured in different levels were significant (P<0.001). Table 3 shows the pairwise comparisons of the mean difference of horizontal and longitudinal dimensions measured in different levels.

**Table 3.** Pairwise comparison of the P-values of the mean width and length according to different levels

**Dimension**		**Upper level**	**Middle level**	**Lower level**
**Horizontal**	Upper level	-	0.253	0.505
	Middle level	-	-	0.634
	Lower level	-	-	-
**Longitudinal**	Upper level	-	<0.001	<0.001
	Middle level	-	-	0.382
	Lower level	-	-	-

In term of horizontal dimensions, there were significant differences between the upper level with middle and lower levels (P<0.05). However, there were no significant differences between the middle and lower levels (P>0.05). Upper level showed the least dimensional changes while the lower level showed the most dimensional changes.

### Interaction effect of level and position on the accuracy of horizontal and longitudinal dimensions:

In terms of horizontal dimension, the effect of level on the accuracy of measurements was not significant (P= 0.41, F= 0.89). However, the effect of position (P< 0.0001, F=22.46), and the interaction effect of level and position (P<0.0001, F=7.63) on the accuracy of measurements was significant. According to the results, central position in the upper level was considered as the most accurate location. Most of the errors occurred at the lower part of the anterior region. In term of longitudinal dimension, the effect of level, position (P< 0.0001, F=21.50), and the interaction effect of level and position (P<0.0001, F=18.11) on the accuracy of measurements were significant (P= 0.41, F= 7.94). According to the results, central position in the upper level was considered to be the most accurate location. Most of the errors occurred at the lower part of the posterior and left regions. In addition, the errors which occurred between the horizontal and longitudinal dimensions were not significantly different (P=0.38, t=0.87).

## CONCLUSION

CBCT application has greatly increased in different fields of dentistry and it has created a revolution in maxillofacial imaging. Its applications include implant surgery, temporomandibular joint imaging, endodontic and orthodontic procedures, evaluation of craniofacial structures and jaw lesions and analysis of airway structures [8]. A previous study compared the accuracy of CBCT and CT and reported greater accuracy of CBCT [27]. However, another study supported the application of CT [12].

In treatment planning for implant surgery, CBCT has some advantages over CT due to its higher spatial resolution in longitudinal dimension, lower radiation dose and lower cost [28]. However, application of CBCT has some limitations including lower signal to noise ratio, lack of accurate determination of Hounsfield unit and poor soft tissue contrast [4]. Since the introduction of CBCT, a large number of studies have focused on the geometric accuracy of this technique [12]. Some factors such as exposure parameters (mAs and kVp), size and position of the FOV, and pixel size can influence the accuracy and quality of CBCT images [2, 21]. FOV refers to the scan volume of a particular CBCT unit, and is the extent to which the beam can cover the area. Shape of the FOV may be either cylindrical or spherical, and one object can be placed in different spatial positions in the FOV [4].

This study aimed to evaluate the dimensional changes of objects at different spatial locations across the FOV. For this purpose, a cylindrical phantom of base-plate wax was constructed to simulate the FOV spatial shape. To mimic the placement of object at different locations in the FOV, aluminum squares with a diameter of 10 mm were placed in the cylinder.

To evaluate the impact of CBCT systems on the accuracy of measurements, phantom was scanned with three different systems and three maxillofacial radiologists evaluated the reconstructed images three times; this minimized measurement errors and allowed the establishment of means and standard deviations. Most of our measurements were underestimated (less than 10 mm) and just a few measurements were equal to the gold standard. In this regard, other studies reported similar results [8,19,20]. Barlik et al. [8] showed that 94.4% of their measurements were underestimated, and these results were in accordance with those of Mozzo et al, [20] who reported that the software tends to underestimate the size of circular objects. Lascala et al. [19] reported that, although the NewTom images underestimate the actual distances between the skull sites, differences are only significant for the skull base and therefore it is reliable for linear measurements of other structures [19].

Pinsky et al. [27] also reported that in I-CAT CBCT system, the phantom measurements had a trend towards underestimation. However, they were not clinically significant. Marmulla et al. [18] found similar results. In most of the afore mentioned reports, the accuracy of CBCT measurements was assessed using human dry skull; with this method, we can compare the measurements obtained from CBCT and digital caliper. However, this method has some demerits: first, the skull morphology was not standardized and a small gap measured between the multi planar reformatting in a CT images and actual values measured with digital caliber, which can lead to large errors. Second, the margins of skulls were determined on the monitor of a personal computer and their accuracy might be influenced by the interobserver and/or intraobserver agreement.

Therefore, we used aluminum inserts with precise dimensions and minimal thickness. Density of aluminum is similar to that of enamel and is greater than that of cortical bone and thus, could produce inconsiderable amounts of artifact [9]. Similarly, we found that all measurements in different positions and systems were underestimated (equal or less than 0.4mm) compared to the actual values. It can be attributed to the systematic errors occurred in CBCT systems. Small scale measurements can lead to significant differences compared to large scale measurements [8].

Yoshida et al. [28] assessed the effect of direction on the measurement accuracy of image using micro-CT. They used a dry mandible with six titanium implants and found more accuracy in the horizontal dimension. They stated that this difference between the horizontal and longitudinal dimensions could be due to the effect of cone angle and insufficient correction by the device in longitudinal dimensions. Tsutsumi et al. [9] used the aluminum phantom and found excellent accuracy in longitudinal dimension. However, the accuracy of horizontal dimension was medium, which could be due to the effect of radiation angle and insufficient correction by the device in horizontal dimension. In contrast, our results showed that there was no significant difference in the amount of errors occurred between the horizontal and longitudinal dimensions. These differences between their results and ours could be ascribed to the different systems used.

In relation to the impact of the object position in the FOV, Tsutsumi et al. [9] scanned the phantom in central and peripheral positions using CB Mercury device. In the longitudinal dimension, there was no significant difference between central and peripheral positions, and longitudinal dimension was not influenced by the position of object in the FOV. However, in the horizontal dimension, there was a significant difference between the central and peripheral position and the central position was considered as the most accurate position.

In the present study, we evaluated the dimensional changes in different positions of the FOV (central, anterior, posterior, left and right) and at three levels (upper, middle and lower). In terms of different positions in horizontal and longitudinal dimensions, there was a significant difference between the central and peripheral positions, and central position showed the least dimensional changes. There was no significant difference between different levels in horizontal dimension. But in longitudinal dimension, there was a significant difference between the upper and other (middle and lower) levels; the upper level showed the least dimensional changes. It seemed that in horizontal dimension, the accuracy of measurement decreased from the upper to the lower level.

The interaction effect of level and position on the accuracy of measurement was significant in horizontal and longitudinal dimensions, and the central position in the upper level was considered as the most accurate position in both dimensions. According to different systems, no significant difference was observed in horizontal dimension. But in longitudinal dimension, there was a significant difference between NewTom and Planmeca devices. This difference can be due to different factors in two systems, such as the pixel size, the exposure conditions, type of detector, geometry settings, and calibration methods.

White and Pharaoh [4] stated that the focal spot size and geometric configuration of the X-ray source are important to determine the degree of geometric unsharpness, a limiting factor in spatial resolution. Geometric setting includes the distance between the x-ray sources and object and the distance between the object to the detector.

In this study, we used the same focal spot size for all devices. Thus, the object position relative to the x-ray radiation source and detector was the only effective variable in the geometric resolution of image.

At every rotation of the x-ray tube in the CBCT unit, different spatial points with different distances related to the tube and detectors are placed in the FOV. The number of individual projection frames may range from 100 to more than 600, to reconstruct volumetric data. In the reconstruction process, reconstruction algorithms evaluate the position of the object in the FOV area and estimate the effective distances in geometric accuracy; this can lead to reporting the exact dimensions of the object. Once all the slices have been reconstructed, they can be recombined into a single volume for visualization.

Probably, the reconstruction algorithm is based on the distance from the center of the FOV, and thus, the accuracy of the reconstruction algorithms for objects in the center of the FOV has been higher. In the current study, the maximum dimensional changes for the object located in different spatial positions and in different systems were 0.4 mm. These findings are clinically important if the dimensional changes are more than ±1mm.

## CONCLUSION

Since the object can be placed in different spatial positions in the FOV, evaluation of the effect of this factor on the dimensional changes is an important issue. We found that the measured values tend to underestimate by less than 0.5 mm. While the difference was statistically significant, it was not clinically significant. Thus, we may conclude that CBCT is an accurate device for measuring the dimensions of an object placed in different spatial positions in the FOV. Central position had the least dimensional changes in both dimensions. Additionally, the highest accuracy was found at the upper level of the FOV.
